# Integrated e-Learning for Shoulder Anatomy and Clinical Examination Skills in First-Year Medical Students: Randomized Controlled Trial

**DOI:** 10.2196/62666

**Published:** 2025-09-17

**Authors:** Roland Koch, Lena Gassner, Navina Gerlach, Teresa Festl-Wietek, Bernhard Hirt, Stefanie Joos, Thomas Shiozawa

**Affiliations:** 1 Institute for General Practice and Interprofessional Care, University Hospital Tübingen, Osianderstr. 5, Tübingen, 72076, Germany, 49 1758065961; 2 TIME - Tuebingen Institute for Medical Education, Medical Faculty, University of Tuebingen, Tübingen, Germany; 3 Department of Anatomy, Institute of Clinical Anatomy and Cell Analysis, Faculty of Medicine, Eberhard Karls University of Tübingen, Tübingen, Germany

**Keywords:** functional anatomy, integrated learning, blended learning, undergraduate medical education, clinical examination, randomized controlled trial

## Abstract

**Background:**

Applying functional anatomy to clinical examination techniques in shoulder examination is challenging for physicians at all learning stages. Anatomy teaching has shifted toward a more function-oriented approach and has increasingly incorporated e-learning. There is limited evidence on whether the integrated teaching of professionalism, clinical examination technique, and functional anatomy via e-learning is effective.

**Objective:**

This study aimed to investigate the impact of an integrated blended learning course on the ability of first-year medical students to perform a shoulder examination on healthy volunteers.

**Methods:**

Based on Kolb’s experiential learning theory, we designed a course on shoulder anatomy and clinical examination techniques that integrates preclinical and clinical content across all 4 stages of Kolb’s learning cycle. The study is a randomized, observer-blinded controlled trial involving first-year medical students who are assigned to one of two groups. Both groups participated in blended learning courses; however, the intervention group’s course combined clinical examination, anatomy, and professional behavior and included a peer-assisted practice session as well as a flipped classroom seminar. The control group’s course combined an online lecture with self-study and self-examination. After completing the course, participants uploaded a video of their shoulder examination. The videos were scored by 2 blinded raters using a standardized examination checklist with a total score of 40.

**Results:**

Thirty-eight medical students were included from the 80 participants needed based on the power calculation. Seventeen intervention and 14 control students completed the 3-week study. The intervention group students scored a mean of 34.71 (SD 1.99). The control students scored a mean of 29.43 (SD 5.13). The difference of means was 5.3 points and proved to be statistically significant (*P*<.001; 2-sided Mann-Whitney *U* test).

**Conclusions:**

The study shows that anatomy, professional behavior, and clinical examination skills can also be taught in an integrated blended learning approach. For first-year medical students, this approach proved more effective than online lectures and self-study.

## Introduction

Although clinical guidelines recommend that imaging diagnostics be based on a structured clinical examination, primary care physicians frequently rely on imaging modalities such as magnetic resonance imaging when managing shoulder complaints [1-4]. This raises the broader issue of how well clinical anatomy and musculoskeletal examination skills are integrated and emphasized during medical education and training. In fact, both residents and medical students report substantial learning needs in musculoskeletal examination techniques, except among those with a preexisting interest in musculoskeletal specialties [5-11].

Learning clinical competencies is a complex process that can be better understood through the application of a theoretical framework [12-14]. Kolb’s experiential learning theory provides such a framework. It conceptualizes learning as a continuous cycle involving concrete experience, reflective observation, abstract conceptualization, and active experimentation [15]. This model is particularly relevant in traditional Flexnerian curricula, where preclinical and clinical training are distinctly delineated, with the cultivation of clinical competencies unfolding over the course of several semesters. It encompasses various dimensions—such as interpreting pathology, applying appropriate examination techniques, and demonstrating professional behavior—achieved through diverse instructional methods, all within a stressful learning environment [3,11,16-20].

Due to the fragmented way students encounter clinical skills and related knowledge, anatomy education has increasingly moved toward region- and function-based integration—though this shift remains incomplete [21-25].

The advent of the COVID-19 pandemic, along with its associated restrictions on in-person interaction, accelerated the adoption of digital teaching formats in medical education. This shift aligned with a general trend toward expanding e-learning in both clinical and anatomical instruction [26-36]. Specifically, shoulder examination techniques can be taught either face-to-face or digitally through synchronous and asynchronous methods, with face-to-face formats demonstrating superior outcomes and higher learner acceptance [9,13,14,27,37]. Blended learning approaches, such as the flipped classroom, which combine digital and in-person elements, have gained increased traction since the pandemic by effectively linking clinical and anatomical content [11,25-27,32,33,38-41]. In addition, peer teaching has been shown to enhance students’ confidence in clinical examination skills and facilitate the transfer of anatomical knowledge into clinical practice [42-44].

The optimal timing for introducing clinical skills within anatomy education and how best to align these skills with examination formats remain subjects of debate [11,45-47]. Clinical skills are predominantly assessed through objective structured clinical examinations (OSCEs), which can also be conducted using video recordings [14,16,48,49].

In summary, Flexnerian curricula typically teach and assess anatomical knowledge, clinical skills, and professional behavior separately, using fragmented instructional methods that include both digital and face-to-face formats. These components correspond to distinct stages of Kolb’s experiential learning cycle—for instance, peer teaching facilitates reflective observation and abstract conceptualization [42], while clinical training fosters concrete experience and active experimentation [9,37]. However, Kolb emphasizes that meaningful and deep learning requires progression through all stages of the cycle [15]. This suggests that educational approaches intentionally integrating these phases may lead to improved learning outcomes.

Although previous studies have demonstrated that anatomy and clinical skills can be taught through both face-to-face and digital modalities [36-38,50], there remains a lack of robust empirical evidence supporting integrated teaching approaches that unify preclinical and clinical domains. Our intervention was therefore designed not only to bridge preclinical and clinical content but also to combine multiple instructional methods, aligning with Kolb’s model to support an effective learning process.

This study aimed to assess the effect of this integrated blended-learning course on first-year medical students’ clinical performance, professionalism, and anatomical knowledge.

## Methods

This paper follows the CONSORT (Consolidated Standards of Reporting Trials) guidelines [51].

### Study Design

The study was conducted as a 2-arm, randomized, observer-blinded intervention. The study’s acronym, TraceX, is derived from “TRansfer of AnatomiCal knowledge in the EXamination situation for preclinical medical students.” It was conducted as part of a curricular development project with the same name. The project was funded by the Medical Faculty of Tübingen University Hospital (Universitätsklinikum Tübingen [UKT]). The study protocol underwent an external peer-review process by reviewers not involved in the project. The dean of the faculty and the faculty commission approved the project in 2020.

**Figure 1.**
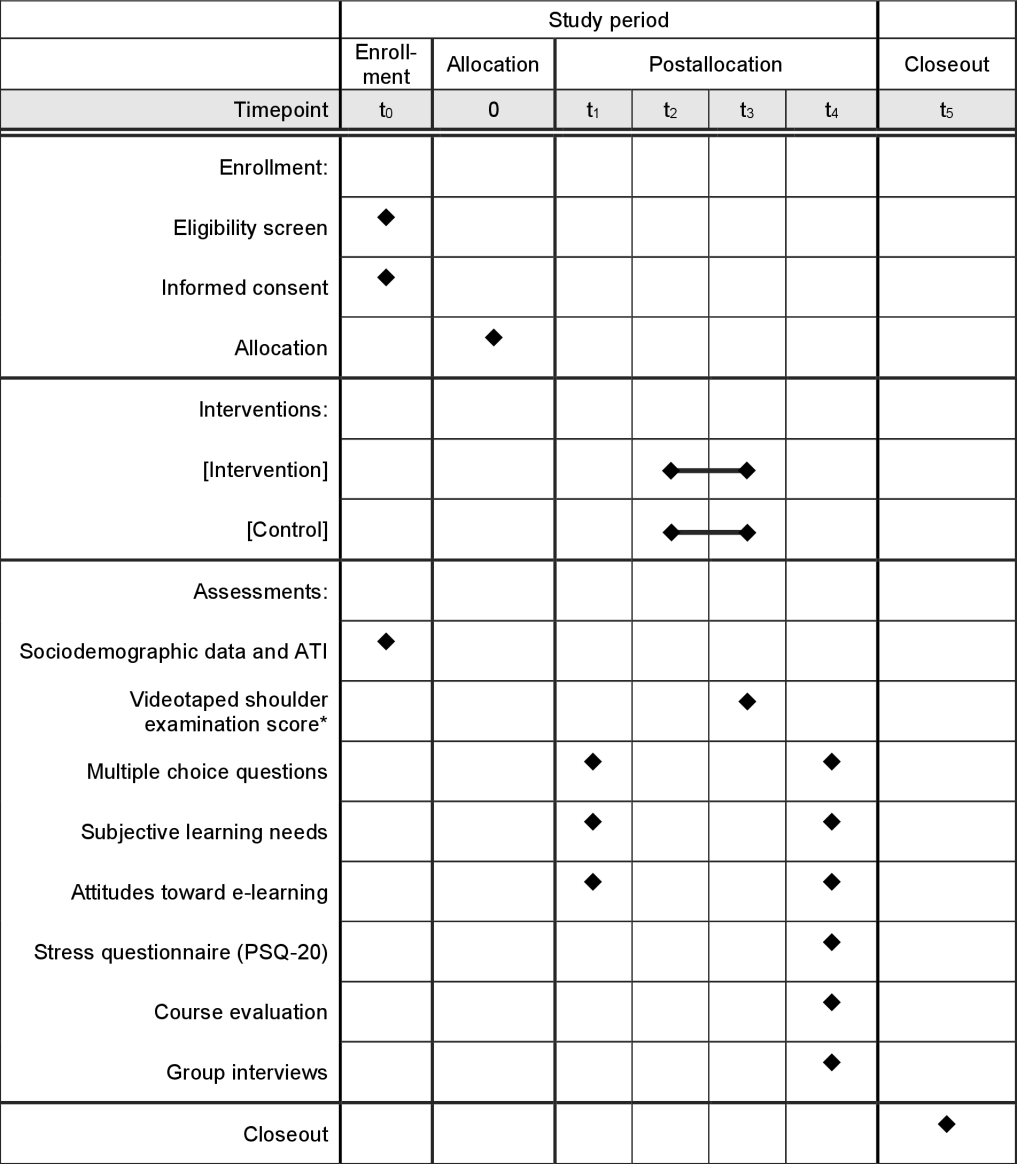
Transfer of anatomical knowledge in the examination situation for preclinical medical students (TraceX) study design protocol. The asterisk indicates the primary outcome. ATI: affinity for technology interaction; PSQ-20: Perceived Stress Questionnaire.

Its protocol is illustrated in Figure 1, based on the SPIRIT (Standard Protocol Items: Recommendations for Interventional Trials) figure template [52]. The study compared students’ performance in a videotaped shoulder examination after a 3-week blended-learning course. The intervention and control groups received 2 different blended-learning modules.

### Study Population and Setting

The study took place at the Medical Faculty of Eberhard Karls University Tübingen in southern Germany. It was conducted between October 2021 and March 2022 by the Institute for General Practice and Interprofessional Health Care in cooperation with the Institute of Clinical Anatomy and Cell Analysis. In Tübingen, online courses are provided using the Integrated Learning, Information, and Work Cooperation System (ILIAS) online-learning platform, which is a commonly used learning management system among German universities (ILIAS open source e-Learning e.V). It allows log-in via authenticated student email accounts.

### Pilot Study

Several instruments used in the study were piloted in 2020 with 18 first-year medical students. The primary purpose of the pilot study was to check interrater reliability for the primary outcome measurement instrument and to calculate the internal reliability of the self-developed questionnaire items. The results of these checks and their impact on the development of the items are listed below for each respective instrument.

In addition, participants of the pilot study were asked to assess the online learning modules. Based on their feedback, the course content was adapted. Two of the pilot study participants provided valuable comments on course benefits and weaknesses in an additional voluntary online interview. The interview served as an extended evaluation. Furthermore, LG (who later conducted the group interviews in the main study) piloted interview guidelines and received interviewer training based on the participants’ feedback.

### Recruitment

Recruitment for the main study started in May 2021. First-year medical students were approached via email and in 2 online lectures. Interested students were invited to contact the Institute for General Practice’s office to receive additional information (eg, type of study, goal, significance, and participant rights). Study participation was voluntary and did not impact other courses in the curriculum. For students who did not wish to participate in the study, regular curriculum activities proceeded. The teaching coordinator (responsible for course and lecture coordination at the Institute) communicated with students but did not participate in the study design or execution. Eligibility criteria included being a first-year medical student, having provided informed consent to use ILIAS, and feeling healthy enough to participate in the study.

### Intervention and Control

Intervention and control courses were developed collaboratively in interdisciplinary workshops involving general practitioners (GPs), a medical didactics expert, an orthopedic surgeon, a psychotherapist, medical students, a physiotherapist, and a medical psychologist.

Common learning objectives were developed first and then applied to both groups, which differed in instructional design and learning format. The intervention group received access to structured online modules, including physiotherapist-led examination videos, functional and topographic anatomy videos performed by an anatomy lecturer, and modules on daily life impact and professional conduct. Content was integrated across preclinical and clinical domains. After self-study, students joined a 90-minute flipped-classroom seminar (taught by the same anatomy lecturer and a GP), which included preparation for a peer-training session. Due to COVID-19 restrictions, this 90-minute peer-training session was held in decentralized student pairs or moderated small groups on Zoom (Zoom Communications Inc), with trained peer tutors facilitating feedback.

The control group followed a more traditional format with lectures and self-study. Students received literature for self-study and instructions for self-examination (eg, palpation in front of a mirror). In addition, they attended 2 distinct synchronous online lectures: one on anatomy (anatomy lecturer) and one on clinical examination (GP), based on the same content as the intervention videos but presented with still images, self-demonstration by the lecturers, and a greater separation of clinical and preclinical content.

**Figure 2.**
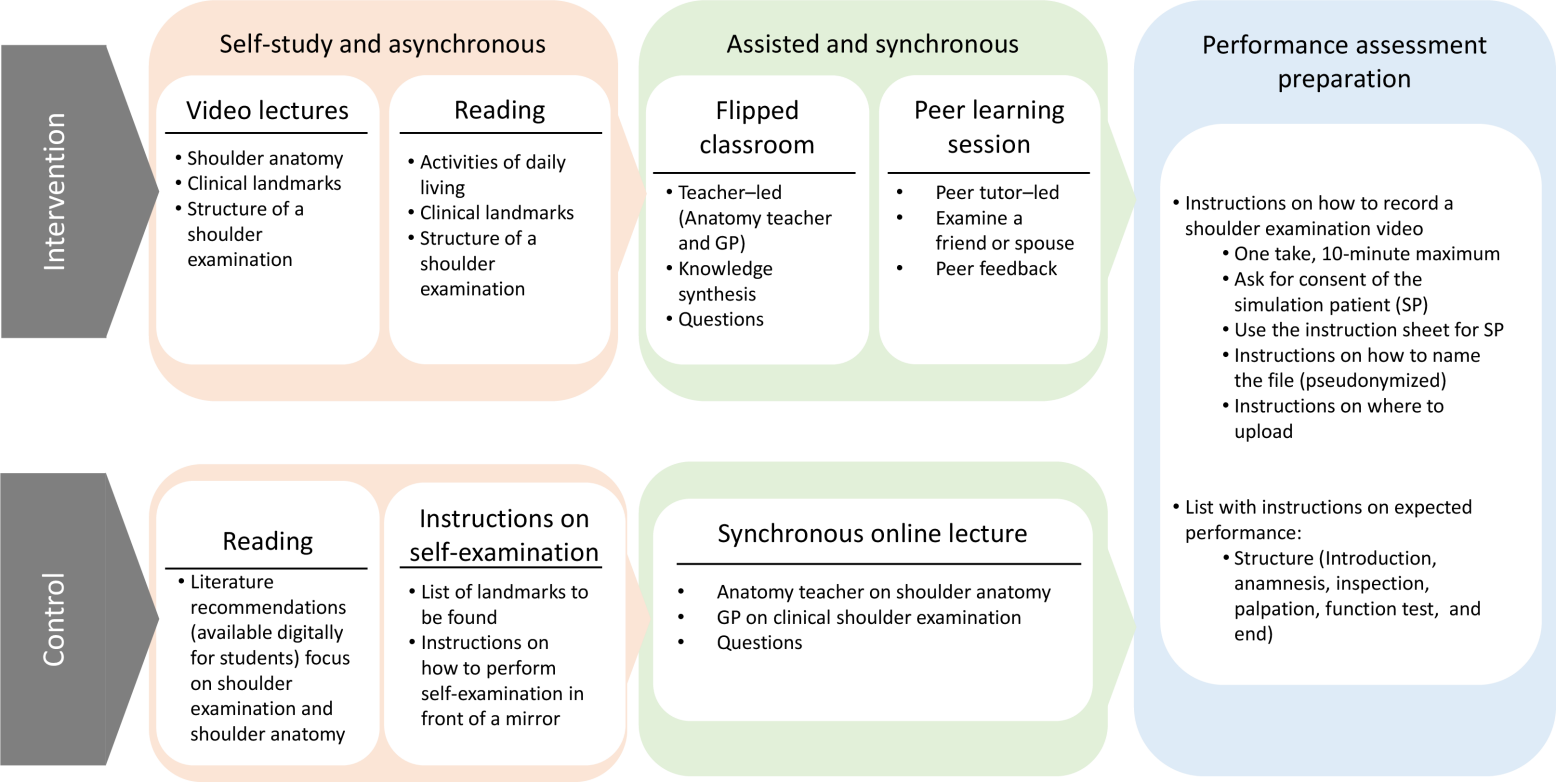
Intervention and control design. GP: general practitioner.

In summary, the intervention emphasized integrated, interactive learning with structured feedback and more practice time, providing a learning process with all 4 steps of Kolb’s learning cycle [15], while the control group reflected conventional, lecture-based learning with limited interaction and a more fragmented learning process. Please refer to Figure 2 for an overview.

### Randomization and Allocation

From those students who signed a written informed consent to participate in the study, basic sociodemographic data were obtained at t0 to enable stratified randomization. Randomization was undertaken in a 1:1 ratio using lists generated with the R programming package (The R Core Team, R Foundation, and R Consortium). Randomization was stratified for gender and occupational experience and used block sizes of 2 and 4. Individualized identification codes were generated by the participants in the surveys (t1 and t4) to allow for linking of presurvey and postsurvey data without compromising participant anonymity or disclosing group affiliation. All other data that could reveal group affiliation were concealed until grading of the final examinations and statistical analysis was completed. Participants were not blinded to their own group affiliation. Randomization and group allocation were performed by 2 researchers not involved in the assessment and development of course content.

The groups received the same instructions for exam performance and grading criteria. Finally, a postcourse questionnaire was distributed to both groups, and voluntary postcourse online group interviews were conducted (t4). After study completion, members of the control group were provided full access to all e-learning resources to preclude disadvantage.

### Primary Outcome

This study hypothesizes that an integrated course based on Kolb’s learning theory would improve first-year medical students’ performance in a standardized, complex OSCE shoulder station requiring structured clinical examination, anatomical knowledge, and professional conduct. Students in the intervention group attended the integrated course and were expected to outperform those in the control group. The primary outcome was OSCE performance at t3, assessed through a structured examination focusing on inspection, palpation, and functional movement assessment, with emphasis on identifying anatomical landmarks rather than detecting pathology.

Because contact restrictions prevented a face-to-face OSCE setting, participants recorded videos of their shoulder examinations and uploaded them to ILIAS. Video length was limited to 10 minutes and included a volunteer (peer, spouse, or family member) acting as a simulation patient. Students were instructed to ensure their volunteers had no preexisting shoulder pain or functional impairments and to obtain consent before recording. Simulation patients were asked to follow the students’ instructions and cooperate during the examination, creating a controlled environment intended to minimize bias introduced by the simulation patients.

The uploaded videos were assessed independently by a pair of blinded raters (GP and a medical student) using an examination checklist. The checklist comprised a total of 40 items, including 12 items on anatomical knowledge, such as topography and functional anatomy, 17 items on structured clinical examination skills, and 9 items on medical professionalism. It was developed in multidisciplinary workshops based on existing literature [14] and items from the OSCE used at UKT [53]. It was piloted in the pilot study. In that study, the pair of most congruent raters (a medical student and a GP) achieved an interrater validity of κ=0.573 (Cohen κ). Due to this moderate agreement, several items were operationalized for better standardization of examiners [54]. The final version of the examination score used in this study is included as Multimedia Appendix 1.

### Secondary Outcomes

Participants’ subjective learning needs were assessed across three domains—anatomical knowledge, clinical examination skills, and professionalism—at time points t1 and t4 using a questionnaire. This questionnaire was developed during an interdisciplinary workshop and subsequently piloted in the initial study phase. The sample of 18 pilot study participants showed a Cronbach α of 0.92. The domain “anatomical knowledge” contained 5 items (ie, visible landmarks, anatomical structure of the shoulder, function, range of motion, and association of function with activities of daily living). The domain “clinical examination” also consisted of 5 items (ie, structured examination steps, history taking, shoulder inspection, shoulder palpation, and test of function). Finally, the domain “professionalism” was covered with 4 items (ie, use of patient-centered language, autonomous handling of the examination situation, perceptiveness toward patient feedback, and addressing patient feedback during the examination). All items were operationalized on a 4-point Likert scale. This yielded sum scores on a 5- to 20-point range and a 4- to 16-point range, respectively.

Theoretical anatomical knowledge was assessed before and after the course (at t1 and t4) using the same set of 10 randomized multiple-choice questions (MCQs). Each question had 5 answer options, with only 1 correct answer, resulting in a knowledge score ranging from 0 to 10. These MCQs reflect the current standard used in the written state medical examination in Germany.

Using Likert scale rating questions, we used 4 items of the standardized Affinity for Technology Interaction Short Scale (ATI-S), rendering a sum score on a 4- to 24-point scale. The ATI-S is a reliable (Cronbach α 0.88-0.92) and validated instrument [55].

The course evaluation included 5 items adapted from the standard evaluation used by the Medical Faculty of Tübingen, covering overall assessment, contribution to personal learning, curriculum alignment, course structure, and clarity of learning goals. Cronbach α for these items was 0.741 based on pilot study data. The evaluation was conducted online via ILIAS at time point t4. In addition, several items assessing blended learning formats were incorporated at t1 and t4, aiming to assess attitudes toward blended learning before and after the intervention. Students were asked to rate whether blended learning promotes self-directed learning, encourages lesson preparation and review, supports exam readiness, enhances curricular value, enables flexible learning, and increases study satisfaction.

Participants were additionally invited to online group interviews at t4. The interviews were analyzed with thematic analysis [56]. An in-depth mixed methods evaluation that includes the interviews will be published separately.

To evaluate the possible impact of the intervention on perceived stress, the validated and standardized 20-item Perceived Stress Questionnaire (PSQ-20) was applied at t4. It contains 20 items with 4 subscales: worries, tension, joy, and demands. Cronbach α is 0.80-0.86 [57].

### Statistical Analysis

#### General

Data were analyzed using SPSS (version 27; IBM Corp). Summary statistics on participants’ demographic characteristics, baseline information, course evaluation, and primary outcome were computed using means, SDs, and proportions.

The following statistical tests were used to assess group differences in primary and secondary outcomes. For the primary outcome and exploratory analysis of subscores, 2-sided Mann-Whitney *U* tests were performed to account for nonparametric data distributions. For comparison between groups for nominal scales, chi-square (χ²) tests were performed or, if cell size was less than 5, Fisher exact test. For the secondary outcomes on longitudinal effects preintervention and postintervention, Wilcoxon rank tests were performed.

Concerning the internal reliability of questionnaires and evaluation based on the pilot study data, Cronbach α was calculated. We also calculated κ statistics underlying the primary outcome for assessment of interrater reliability in examination grading.

The study’s intention-to-treat approach considers possible contamination effects by between-group communication or student account misuse between groups.

#### Power

Power analysis suggested a sample size of 62 subjects to detect intergroup differences in examination scores of 16% with a SD of 20% and an estimated effect size of 0.71 when using 2-sided *t* tests for independent samples at a 5% significance level [58]. Adding a 25% buffer for dropouts, a total of 80 participants were aimed for as the total sample size.

### Ethical Considerations

The study was assessed by the Ethics Committee of the Medical Faculty of Tübingen University on May 4, 2020, (231/2020BO1) and did not require ethical approval. Study participation was voluntary and students provided informed consent to participate in the study and access the respective course material in ILIAS. No financial inducement was given for study participation. However, all participating students received a bookstore voucher to compensate for their time commitment. Furthermore, participants who completed the study requirements were eligible to enter a lottery drawing for one iPad. Data were stored on ISO 27001–certified servers at UKT. The trial protocol is available from the authors upon request.

## Results

### Study Flow

**Figure 3.**
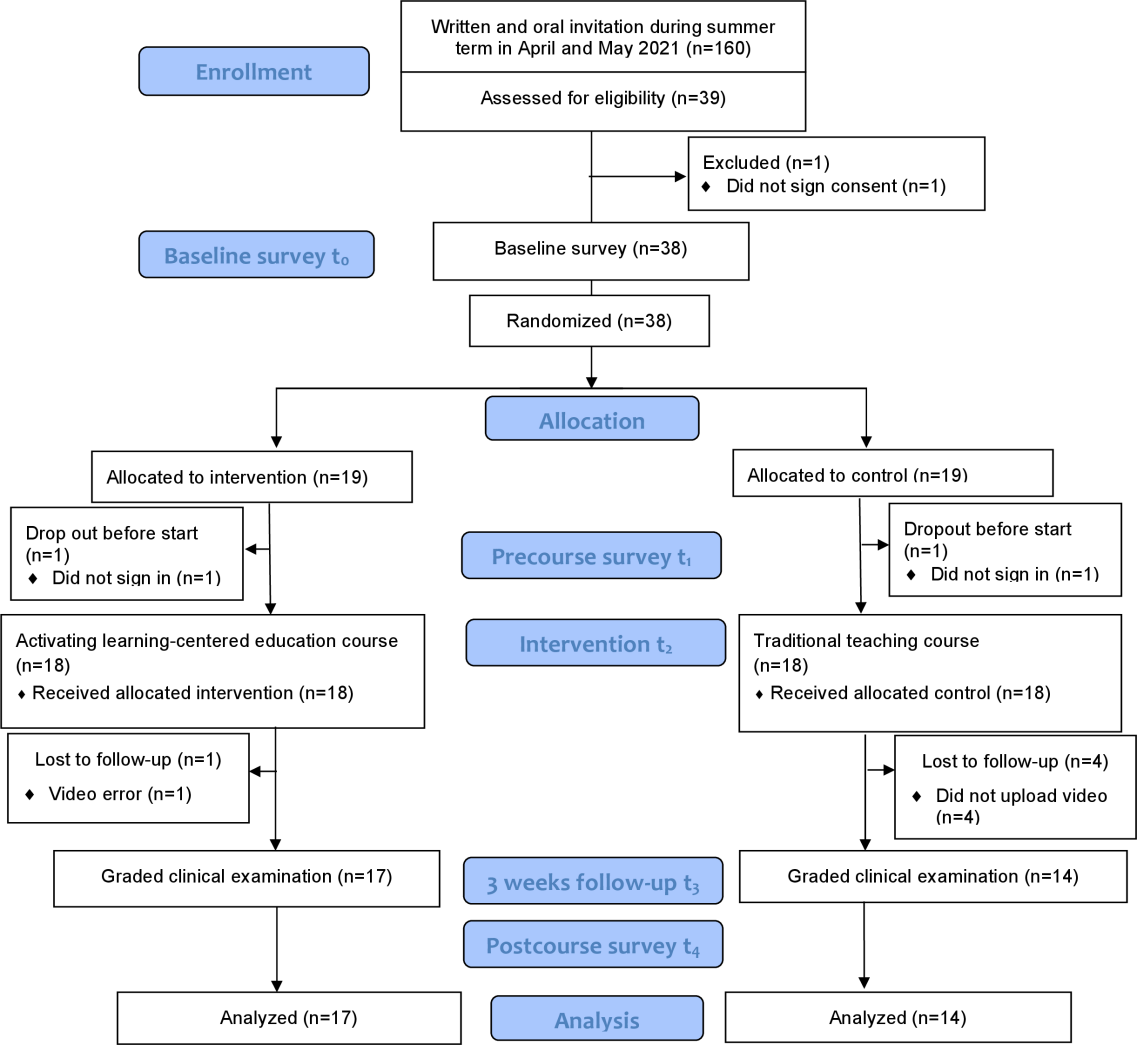
CONSORT (Consolidated Standards of Reporting Trials) study flowchart.

Of 39 enrolled students, 38 were allocated to the intervention and control groups. In both groups, one participant, who, despite having signed informed consent, did not sign in to ILIAS. In the intervention group, one student dropped out before analysis due to an unresolvable video error. The control group had 4 dropouts who did not upload a video. In total, analyzable data from 31 study participants were obtained. Figure 3 below shows the study flow (CONSORT chart).

### Sociodemographic and Occupational Characteristics

The majority of participants had no current employment. The intervention group had 2 more participants currently employed in the medical sector. Statistically, neither current nor past employment differed significantly between groups. Participant characteristics at baseline (t1) are presented in Table 1.

**Table 1.** Participant characteristics (N=18).

Characteristic	Intervention	Control	*P* value
Age (years), mean (SD)	21.78 (3.56)	21.94 (5.68)	.92^a^
Female^b^, n (%)	13 (72)	14 (78)	≥.99^c^
Previous occupational experience, n (%)			
Yes	9 (50)	8 (44)	.74^d^
Currently employed, n (%)			
Yes, medical sector	4 (22)	2 (11)	.73^c^
Yes, nonmedical sector	1 (6)	2 (11)	—^e^
No	13 (72)	13 (72)	—
Missing	0 (0)	1 (6)	—

a Student *t* test.

b No participant identified as nonbinary.

c Fisher exact test.

d Chi-square test.

e Not applicable.

### Primary Outcome

#### Overview

**Figure 4.**
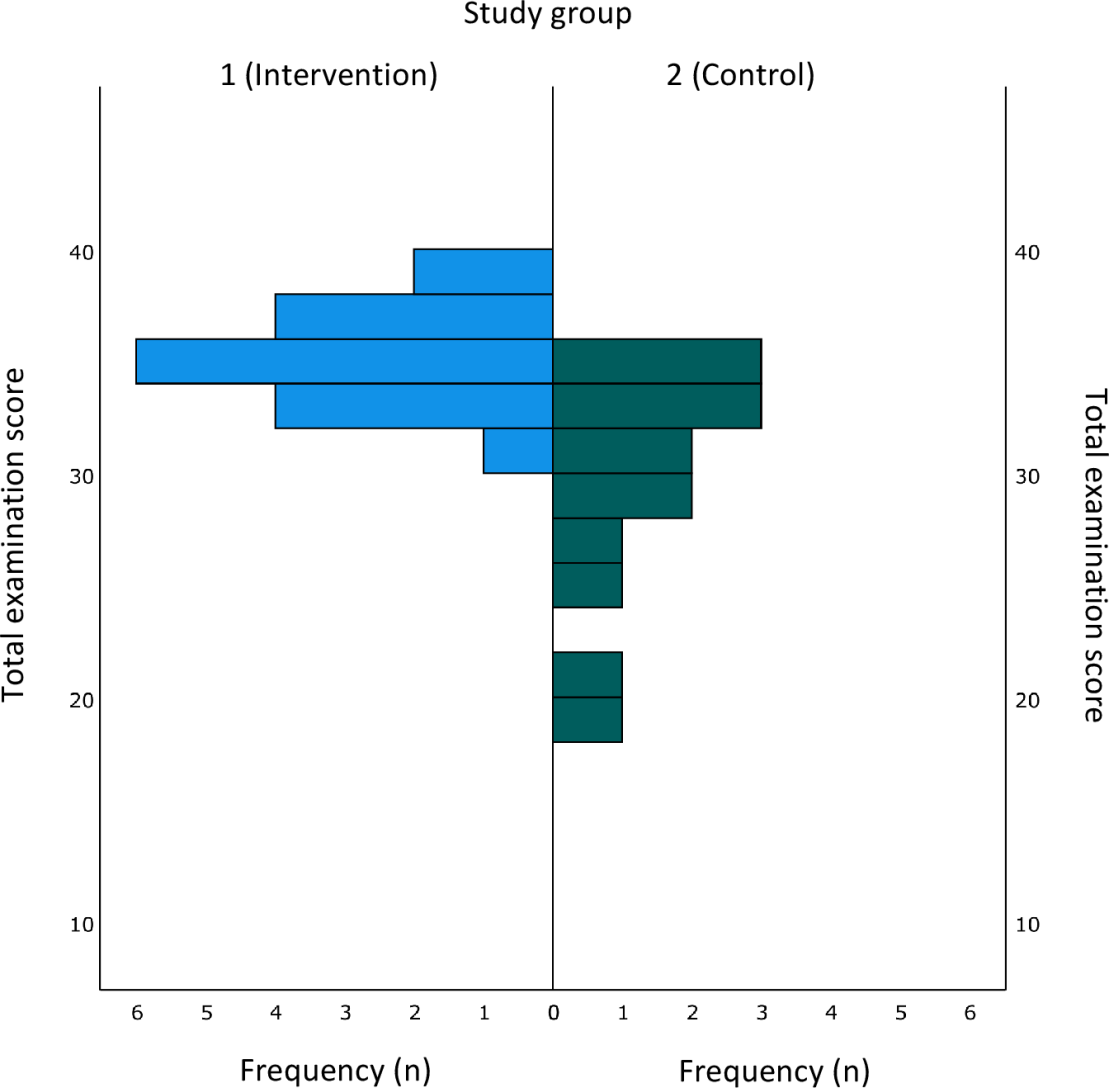
Distribution of sum scores in the graded examination per group.

Intervention group participants reached higher mean performance scores compared to those in the control group (mean 34.71, SD 1.99 vs mean 29.43, SD 5.13), with differences being statistically significant at *P*<.001. Score distributions are shown in Figure 4. Interrater reliability for the shoulder examination checklist score between the two blinded raters was 0.763.

#### Exploratory Analysis of Primary Outcome Subscores

We conducted a post hoc exploratory analysis of the primary outcome subscores: anatomical knowledge, clinical examination skills, and professionalism. In the anatomy subscore, the intervention group performed slightly better than the control group, with the difference reaching statistical significance. For clinical examination skills, the difference between groups was less pronounced but remained statistically significant. Table 2 summarizes the results of this analysis.

**Table 2.** Exploratory analysis of primary outcome subscores.

Variable	Intervention, mean (SD)	Control, mean (SD)	*P* value^a^
Total performance score (40 items, primary outcome)	34.71 (1.99)	29.43 (5.13)	<.001
Anatomical knowledge (12-item subscore)	9.88 (1.73)	8.21 (1.93)	.02
Clinical examination (17-item subscore)	17.88 (0.86)	15.14 (3.92)	.05
Medical professionalism (9-item subscore)	6.94 (0.90)	6.07 (1.44)	.15

a Mann-Whitney *U *test, exact significance.

### Secondary Outcomes

Both groups showed a significant reduction in self-reported learning needs in anatomy and clinical examination. MCQ performance improved significantly in both groups after the course, with no significant differences between groups. Participants in the intervention group spent, on average, 2 hours more in the learning module than those in the control group, a statistically significant difference. No relevant group differences were found in attitudes toward blended learning. In the course evaluation, the intervention group rated the blended learning experience and overall course evaluation less favorably but gave higher ratings for the achievement of learning goals and curricular alignment compared to the control group.

Table 3 provides an overview of the secondary outcomes. Group differences at t1 were not significant and are not included.

**Table 3.** Secondary outcomes.

Measurement	Dropout (n=5), t_1_, mean (SD)	Intervention (n=17), t_1_, mean (SD)	Intervention, mean (SD)	Difference, t_4_- t_1_	*P* value^a^ (interv)	Control (n=14), t_1_, mean (SD)	Control, t_4_, mean (SD)	Difference, t_4_- t_1_	*P* value^a^ (control)	Difference intervention-control (time); positive: favors intervention	*P* value^b^
Affinity for technology interaction	11.2 (4.55)	12.65 (3.52)	—^c^	—	—	14 (2.96)	—	—	—	−1.35 (t_1_)	.36
Time spent (hours)	—	—	6.06 (2.42)	—	—	—	4.0 (1.76)	—	—	2.06 (t_4_)	.01
Perceived Stress Questionnaire score, index	—	—	0.46 (0.09)	—	—	—	0.47 (0.11)	—	—	−0.01 (t_4_)	.98
Learning needs											
Subscore anatomy	17.2 (2.17)	15.76 (2.73)	9.53 (2.27)	−6.24	.00	15.57 (3.8)	10.43 (2.06)	−5.14	.00	−0.90 (t_4_)	.22
Subscore professional conduct	11.6 (3.78)	8.76 (2.75)	6.82 (2.43)	−1.94	.00	9.5 (4.07)	8.07 (2.06)	−1.43	.13	−1.25 (t_4_)	.19
Subscore examination skills	19 (1.73)	18.41 (1.77)	9 (2.35)	−9.41	.00	18.43 (3.52)	11.5 (2.47)	−6.93	.00	−2.50 (t_4_)	.01
MCQs^e^	4.6 (2.07)	5.53 (2)	6.94 (1.43)	1.41	.01	4.71 (2.09)	6.5 (1.83)	1.79	.00	0.82 (t_4_)	.63
Attitudes toward blended learning (mean item score; 1=strongly disagree, 5=strongly agree)											
Facilitate continuous self-sufficient learning	2.4 (1.14)	2.76 (1.2)	2.65 (1.32)	−0.12	.56	2.93 (0.73)	2.79 (0.89)	−0.14	.63	−0.14 (t_4_)	.68
Motivate me to prepare for or follow up on classroom events	2.8 (1.3)	2.94 (0.93)	2.94 (1.02)	0.00	1	2.93 (0.83)	2.57 (0.51)	−0.36	.17	0.37 (t_4_)	.32
Support me in preparing for exams	2 (1.23)	2.65 (0.93)	2.59 (0.8)	−0.06	.76	2.50 (0.86)	2.29 (0.61)	−0.21	.37	0.3 (t_4_)	.23
Provide added value in learning	4.2 (1.1)	3.35 (1)	3.47 (1.07)	0.12	.6	3.43 (0.76)	3.36 (0.63)	−0.07	.74	0.11 (t_4_)	.92
Allow learning at any time and from any place	1.2 (0.45)	1.59 (0.87)	1.53 (0.87)	−0.06	.65	1.14 (0.36)	1.86 (0.86)	0.72	.01	−0.33 (t_4_)	.22
Increase satisfaction with my studies	2.2 (1.3)	3.18 (1.07)	2.71 (1.26)	−0.47	.05	3.57 (0.85)	3.14 (0.66)	−0.43	.11	−0.43 (t_4_)	.22
Facilitate my learning	2.2 (1.1)	2.76 (1.25)	2.47 (1.18)	−0.29	.06	2.71 (0.83)	3.07 (0.73	0.36	.21	−0.6 (t_4_)	.11
Evaluation (mean item score; 1=strongly disagree, 5=strongly agree)											
The blended learning module was well executed	—	—	1.94 (0.83)	—	—	—	2.86 (0.66)	—	—	−0.92 (t_4_)	.01
Learning goals were clearly defined	—	—	4.24 (0.56)	—	—	—	3.21 (0.8)	—	—	1.03 (t_4_)	.00
The course was clearly structured	—	—	4.59 (0.5)	—	—	—	3.57 (0.94)	—	—	1.02 (t_4_)	.00
The course aligned well with the curriculum	—	—	4.35 (0.6)	—	—	—	3.57 (1.16)	—	—	0.78 (t_4_)	.03
The course contributed to my learning success	—	—	4.06 (0.75)	—	—	—	3.57 (1.1)	—	—	0.49 (t_4_)	.17
Course assessment (grade; 1=very good, 6=insufficient)	—	—	1.88 (0.6)	—	—	—	2.57 (0.85)	—	—	−0.69 (t_4_)	.03

a Wilcoxon rank-sign test.

b Mann-Whitney *U* test.

c Not applicable.

d MCQ: multiple-choice question.

## Discussion

### Principal Findings

#### Overview

This randomized, observer-blinded trial tested whether an integrated course based on Kolb’s learning theory would improve first-year medical students’ OSCE performance. Students in the intervention group outperformed the control group after a 3-week course. Both groups showed reduced learning needs, though intervention students spent approximately 2 hours more in the course. The study confirmed the hypothesis but had limitations to consider.

#### Clinical Examination Skills

The observed difference in clinical examination performance between the 2 groups corresponded to a significant reduction in learning needs related to examination skills. These findings align with previous studies. Brewer et al [37] found that second-year students who received face-to-face instruction in clinical examination skills performed better than those in asynchronous e-learning or self-study groups, with the self-study group achieving the lowest scores. Brewer’s study used simulated patients without pathology and included a broader range of clinical tests. Both Brewer et al [37] and Vivekananda-Schmidt et al [13] reported high effect sizes for OSCE shoulder performance when students supplemented regular studies with asynchronous computer-assisted learning. Vivekananda-Schmidt also noted improved student confidence with computer-assisted learning, though our study did not assess confidence due to its limited relevance to performance [7,12].

#### Anatomical Knowledge

Participants in the intervention group demonstrated improved clinical examination performance without a negative impact on their theoretical anatomical knowledge, as measured by MCQs. We believe that the exclusion of advanced clinical examination tasks such as the Jobe test or Hawkins sign from the learning goals contributed to the increase in anatomical knowledge demonstrated in the exam and the more pronounced reduction of anatomical learning needs in the intervention group. According to the participating preclinical and clinical teachers in the multidisciplinary workshop, these tests require extensive knowledge of clinical pathology and might confuse first-year medical students. This notion is in line with existing research that advises careful didactic planning of anatomical content [2,3,11].

Although the online instruction in our study offered only basic anatomical content—focusing on muscles, bones, and function while underrepresenting coverage of nerves and vessels—both groups had recently attended curricular lectures designed to provide deeper anatomical knowledge. After the course, participants in both groups could identify anatomical structures and assess normal findings in a structured way. The key difference was that the intervention group had the chance to apply their theoretical knowledge in practice and receive structured feedback during the peer tutoring session [11,42]. Our findings suggest that early integration of practical experience does not hinder but may, in fact, support deeper anatomical and clinical learning if implemented appropriately [3,11,25].

#### Professional Conduct

Neither the professional subscore in the shoulder examination nor learning needs showed significant differences between groups. Professional conduct might represent a subjective feeling of confidence rather than a measurable outcome. It may not have been adequately operationalized in this study; for example, the relative passive role of the standardized patients posed no real challenges for professionalism. In addition, too little time was allocated to student-teacher interaction and exchange about professional conduct—a common shortcoming of blended learning [32]. This shows that some aspects of learning should be performed face-to-face and that time must be allocated for this interaction [26,30,32]. Also, professional conduct is difficult to teach explicitly and is often learned from various sources over a long period of time [13,18,19,27]. Social learning elements play a crucial role in fostering professionalism even in a predominantly remote learning environment [32]. It thrives through direct interaction between instructors and learners. This insight will inform the curricular integration of our learning module.

In summary, this study shows that clinical examination skills can successfully be taught early in the curriculum using a blended learning approach. The reduction in learning needs was not dependent on current or past employment or different levels of preexisting anatomical knowledge. We controlled for differences in gender and preoccupation through randomization. A baseline performance check using a structured examination before the study would perhaps have shown a clearer dependence on preexisting examination skills [59].

### Dropout Analysis

The 5 dropouts had higher subjective learning needs at t1, scored lower in the MCQ knowledge test, and had a slightly lower technical affinity. Their attitudes toward blended learning differed from those of the other study participants. While they believed that blended learning provided added value to learning, they were less confident that it helped them prepare for exams or increase personal satisfaction with their studies. The dropout sample was too small for statistical analyses. However, there are 2 ways to interpret this: there might be a subgroup of students with such low expectations of blended learning that they might not use the method if given the choice [60], or the small study population could reflect a selection bias of motivated, stress-resilient, technology-affine, high-performing students who would do well in any learning environment [32].

### Other Secondary Outcomes

The intervention group required, on average, 2 additional hours to complete the course compared to the control group. This was due to engagement with video lectures and demonstrations, as well as participation in the peer-assisted synchronous learning session, all of which required learners to allocate additional time, even though they had greater flexibility to choose when and where to engage with most of the content than the control group. Similar increases in workload have been observed in other studies on blended learning formats [36].

This interpretation is further supported by the intervention group’s high ratings of course structure, clarity of learning objectives, and perceived alignment with the curriculum.

Although overall stress levels were high in both groups at t4, they were comparable to those of other medical student cohorts [20]. We therefore infer that the increased workload did not result in a heightened perception of stress among intervention group participants.

Technical affinity was equally high across both groups, consistent with findings that current medical student cohorts are generally digitally literate [27]. For our sample, this suggests that time invested in studying, rather than digital affinity, was more strongly associated with learning success [46,47].

### Implications for Curricular Development

#### When Should Clinical Aspects in Anatomy Learning Be Integrated?

Our study supports the integration of anatomy teaching and clinical examination early in the curriculum. Both study groups, exposed to clinical examination content during the first year, reported reduced learning needs and demonstrated measurable skill acquisition without compromising anatomical knowledge. These findings suggest that early competency development is feasible and particularly effective when teaching content and assessment formats are well aligned [10,14,21-25,45].

#### What is the Role of Blended Learning in Integrated Clinical and Anatomy Learning?

Our study demonstrated that an intervention using synchronous peer-assisted clinical training paired with asynchronous training videos in a flipped-classroom seminar improved student outcomes compared to self-study and synchronous online lectures, aligning with previous studies [30,37]. However, despite improved performance, students reported a less satisfactory blended learning experience, highlighting the challenge of effectively implementing such approaches [27,29]. Based on our findings, key factors for successful blended learning include clear learning goals, structured delivery, and theory-guided integration of content and teaching methods, which may be more influential than the delivery format itself on student evaluation [32,33]. In addition, effective implementation must account for contextual factors such as the COVID-19 pandemic, which may have influenced student perceptions in our study [26]. These findings highlight that while blended learning can enhance outcomes, its effectiveness depends on thoughtful design, clear objectives, and contextual factors—underscoring the need for nuanced, context-aware research in this field [41].

### Limitations

This study is limited by its small sample size, although a power analysis indicated a need for 80 participants; only 38 were recruited, and 31 completed the trial. While randomization controlled for gender and professional experience, the small sample size limits generalizability and prevents definitive conclusions about causality.

Recruitment was difficult despite incentives, likely due to increased student workload during the COVID-19 pandemic [26,27]. Although results were promising, the recruitment shortfall and end of funding prevented a second cohort. In addition, curriculum changes during the later stages of the pandemic affected the study environment, leading us to conclude the trial and prioritize efforts to integrate the intervention into the curriculum.

Concerning the main outcome, the intervention course provided learners with tailored modular learning material and feedback opportunities, in contrast to the control group. Importantly, both groups received teaching on shoulder anatomy and clinical skills—a more integrated approach than the regular curriculum—and identical guidance on the performance assessment. This substantially reduces the study’s ability to isolate the effect of blended versus traditional learning. However, both groups showed knowledge gains, suggesting that early integration of clinical content is effective overall. The superior performance in the intervention group likely reflects not only the learning format but also the stronger alignment between the instructional design and the assessment tasks—consistent with our hypothesis that an integrated, theory-based course would improve clinical competency [47].

A reconnaissance effect on anatomical knowledge cannot be excluded, as the same MCQs were repeated at t4. However, student performance and learning needs indicated stable or improved knowledge. Peer learning sessions and exams were intended to be conducted face-to-face but were instead held via videoconferencing due to COVID-19. Although this deviated from the protocol, comparable learning outcomes have been reported in other studies. Video-recorded OSCEs are an established method [16], with only one upload failure. Interobserver reliability was substantial and consistent with other studies [9,14,37].

### Conclusions

First-year medical students’ clinical performance on shoulder examinations can objectively be improved by an integrated blended-learning course in anatomy, professional conduct, and clinical examination skills. The results encourage early integration of clinical examination skills in preclinical medical education. Didactically, early clinical examination courses should focus on functional anatomy, a structured examination approach, and healthy subjects. Methodologically, the content can be delivered using blended learning that considers social aspects of learning and aligns the content to a standardized shoulder examination simulating a single OSCE station. If a face-to-face exam is not possible, videotaped examinations are a viable alternative. This study does not provide information about the sustainability of the learning. A cohort study that follows the progression through surface and deep learning phases to the transfer phase in this integrative approach is warranted [11].

## Supplementary material

10.2196/62666 Multimedia Appendix 1 Translation of the checklist used for video upload scoring (primary outcome).

10.2196/62666 Checklist 1 CONSORT (Consolidated Standards of Reporting Trials) checklist.

## Abbreviations

ATI-S: Affinity for Technology Interaction Short Scale
CONSORT: Consolidated Standards of Reporting Trials
GP: general practitioner
ILIAS: Integrated Learning, Information, and Work Cooperation System
MCQ: multiple-choice question
OSCE: objective structured clinical examination
PSQ-20: 20-item Perceived Stress Questionnaire
SPIRIT: Standard Protocol Items: Recommendations for Interventional Trials
TraceX: Transfer of anatomical knowledge in the examination situation for preclinical medical students
UKT: Universitätsklinikum Tübingen
