# Supplementary material for: Integrated e-Learning for Shoulder Anatomy and Clinical Examination Skills in First-Year Medical Students: Randomized Controlled Trial
Source: JMIR Med Educ. 2025 Sep 17;11:e62666. doi: 10.2196/62666 (PMC12443353; doi:10.2196/62666)
Supplement: Multimedia Appendix 1 [file mededu-v11-e62666-s001.docx]

**Assessment Checklist Shoulder Examination TraceX**

| 1. Start and Introduction | Points (max. 8) |
| --- | --- |
| 1.1 Preparation: Hand disinfection, short sleeves, no jewelry.  *Only give a point if everything is visible + hand disinfection is at least implied.* |  |
| 1.2 Greeting, introduction with name and function |  |
| 1.3 Ask patient‘s name |  |
| 1.4 Ask reasons for the patient’s presentation |  |
| 1.5 Explain how the examination will be structured |  |
| 1.6 Ask for symptoms and complaints |  |
| 1.7 Ask for activities of daily living and how symptoms might influence them |  |
| 1.8 Ask patient to take off shirt |  |
| Total points introduction |  |

| 1. Inspection (both sides) | Points (max. 5) |
| --- | --- |
| 2.1 Announces inspection |  |
| 2.2 Inspect both sides  *Shoulder position sufficient for criterion "side comparison". Mandatory: Inspection from front and back.* |  |
| 2.3 Comments on and presents inspection findings to patient |  |
| Findings |  |
| 2.4 Clinical abnormalities of posture/movement, scars, skin type, texture. |  |
| 2.5 Symmetry: Bony structures (clavicle, acromion, scapula), muscle build, shoulder stance.  *Anatomical structures on one side suffice for awarding a point* |  |
| Total points inspection |  |

| 1. Palpation/ Demonstrating Landmarks (both sides) | Points (max. 9) |
| --- | --- |
| 3.1 Announces palpation |  |
| 3.2 Palpates both sides  *Front and back, announcement "I would palpate the other side too” is not enough - touching AND naming is required to be awarded a point* |  |
| 3.3 Comments on and presents palpation findings to patient |  |
| Findings |  |
| 3.4 Clavicle |  |
| 3.5 Acromion and acromioclavicular joint |  |
| 3.6 Processus coracoideus |  |
| 3.7 Deltoid muscle |  |
| 3.8 Scapula and Spinae Scapulae |  |
| 3.9 Trapezius muscle |  |
| Total points palpation |  |

| 1. Active range of motion (symmetrically both sides) | Points (max. 12) |
| --- | --- |
| 4.1 Announces active range of motion testing |  |
| 4.2 Instructs the patient (Verbalizes movements or demonstrates them) |  |
| 4.3 Performs range of motion check on both sides |  |
| 4.4 Comments on and presents range of motion findings to patient |  |
| Befunde |  |
| 4.5 Abduction |  |
| 4.6 Adduction |  |
| 4.7 Anteversion |  |
| 4.8 Retroversion |  |
| 4.9 Internal rotation |  |
| 4.10 External rotation |  |
| 4.11 Asks the patient to place both hands behind their back |  |
| 4.12 Asks the patient to place both hands behind their head |  |
| Total points active range of motion testing |  |

| 1. Summary and ending | Points (max. 1) |
| --- | --- |
| Summarises result, clear conclusion, ends examination  *Summary of result is mandatory, saying good-bye is not mandatory to be awarded a point* |  |
| Total points Summary and ending |  |

| 1. Structure | Points (max. 2) |
| --- | --- |
| 6.1 Correct order of examination  (Introduction - Inspection – Palpation – Range of motion testing – ending) |  |
| 6.2 Moderates examination flow and structure independently  *e.g. comments on the procedure, moderates transitions, prompting* |  |
| Total points structure |  |

| 1. Communication | Points (max. 3) |
| --- | --- |
| 7.1 Patient-adapted speech: open, slow, distinct, appropriate volume |  |
| 7.2 Patient-adapted expression of speech: no (unexplained) medical terms; patient-adapted naming and description of anatomical landmarks |  |
| 7.3 Attentiveness in the examination  *e.g. favourable shaping of the conversation through gestures/ facial expressions, empathetic appearance, eye contact, allowing for excuses* |  |
| Total points communication |  |

| Subscores |  |
| --- | --- |
| Start and Introduction (max. 8) |  |
| Inspection (max. 5) |  |
| Palpation (max. 9) |  |
| Active range of motion testing (max. 12) |  |
| Summary and ending (max. 1) |  |
| Structure (max. 2) |  |
| Communication (max. 3) |  |
| Total score (max. 40) |  |

What was good? [1]

*Free text: note what you thought was well performed*

What could you improve? [1]

*Free text: Note any points for improvement*
